# Dataset from *de novo* transcriptome assembly of *Myristica fatua* leaves using MinION nanopore sequencer

**DOI:** 10.1016/j.dib.2022.108838

**Published:** 2022-12-17

**Authors:** Deden Derajat Matra, M Adrian, Karmanah, Jakty Kusuma, Jérôme Duminil, Sobir, Roedhy Poerwanto

**Affiliations:** a Department of Agronomy and Horticulture, Faculty of Agriculture, IPB University, Bogor, Indonesia; b Agronomy and Horticulture Study Program, Graduate School of IPB University, Bogor, Indonesia; c Agrotechnology Study Program, Faculty of Agriculture, Nusa Bangsa University, Bogor, Indonesia; d Department of Plant Science, Politeknik Negeri Lampung, Lampung, Indonesia; e DIADE, IRD, Univ Montpellier, CIRAD, Montpellier, France

**Keywords:** Conservation, Genetic diversity, Long-reads sequencing, Spices, Full-length transcript

## Abstract

*Myristica fatua* is a tropical fruit tree species originating from Indonesia. Very few genomic resources are available for the species. We developed a full-length transcriptome assembly using long-read sequencing (MinION Nanopore technology) and produced 4.3 million reads (3.5 G of bases). The assembled full-length transcript was constructed using the RATTLE program and assembled 21,098 transcripts. The transcript ranged from 201 – 14,174 bp, and N50 was 2,017 bp. The transcripts were annotated with the UNIPROT database using BlastX. The functional annotation was performed using Blast2go software. The 8,445 microsatellite motif-containing contigs were identified. The raw reads are deposited in the ENA (European Nucleotide Archive) with ENA experiment accession number ERX6798613.

**Specifications Table**

**Table:** 

Subject area	Agronomy and Crop Science
More specific subject area	Spices
Type of data	RNA sequencing Data
How data was acquired	MinION Nanopore Sequencer
Data format	Raw Sequencing reads, Table, Figure
Description of data collection	RNA sequencing was performed by using MinION Nanopore on leaves from the seedling stage
Data source location	Dramaga, Bogor, West Java, Indonesia
Data accessibility	The data have been deposited in the European Nucleotide Archive (ENA) at EMBL-EBI under accession number ERX6798613 (https://www.ebi.ac.uk/ena/browser/view/ERX6798613) or https://identifiers.org/insdc.sra:ERX6798613
Related research article	Kusuma, J., Scarcelli, N., Couderc, M. et al. Microsatellite markers development for Indonesian nutmeg (*Myristica fragrans* Houtt.) and transferability to other Myristicaceae spp.. Mol Biol Rep 47, 4835–4840 (2020). https://doi.org/10.1007/s11033–020–05535-y[1]

## Value of the Data

• This data provides *Myristica fatua* coding sequence (CDS) as the first transcriptome reference using Oxford Nanopore Technologies of long-read sequencing
• This data could benefit studies to identify full-length transcripts related to flavonoid biosynthesis for molecular biologists that are used for downstream analysis in *Myristica fatua* and related genera.
• This data provides datasets of EST-microsatellite molecular markers for the breeder to improve crop breeding programs in *Myristica fatua-*related genera*.*
• The raw sequencing data may be carried out further in differential expressed gene study.

## Objective

This plant is one of the best potential spices sources from Indonesia. However, genetic information such as transcriptome data is not yet available. Therefore, these data were used to obtain transcriptome information from leaves in the seedling phase of *M. fatua*. The transcripts' results were obtained using long-read sequencing from oxford nanopore technology. This data is able to provide full-length transcripts that are useful for studying gene expression analysis.

## 1 Data Description

**Fig. 1:**
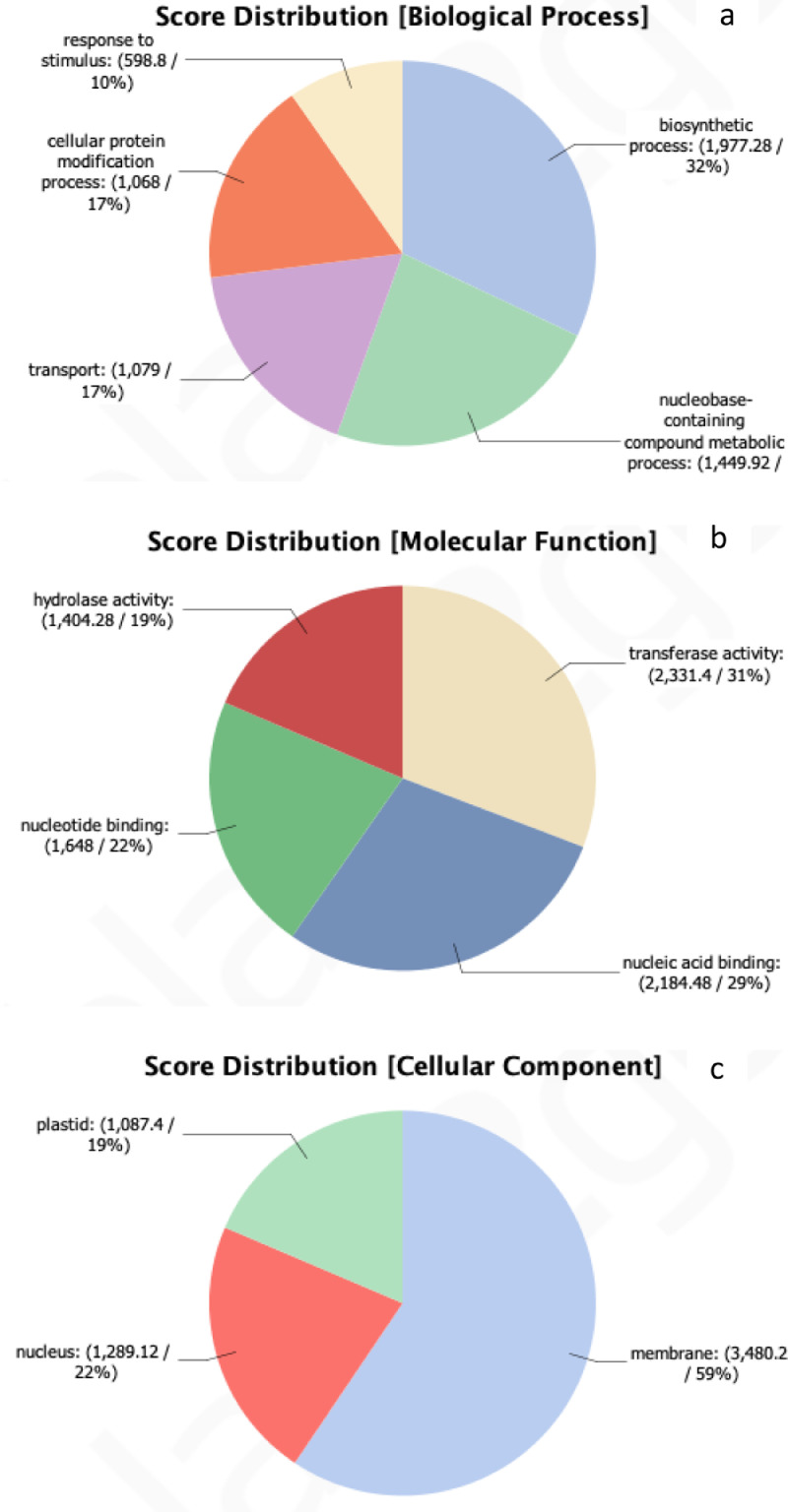
Gene Ontology (GO) classification of *Myristica fatua* for (a) Biological Process, (b) Molecular Function, (c) Cellular Component.

In this data, full-length transcripts were sequenced from *Myristica fatua* using long-read sequencing. The total RNA was extracted from the leaves on the seedling stage with high-quality total RNA. The full length was obtained with raw data produced 4.3 million reads (3.5G of bases) [2]. The raw reads are deposited in the ENA database with the accession number ERX6798613 [3]. The clean reads were filtered by pychopper and cutadapt programs. The de novo assembly was constructed using the RATTLE program and produced 21,098 transcripts [4]. All statistics of reads and assembled transcripts were analyzed (Table 1). The transcripts were annotated with a filtered-UNIPROT database using the BLAST+ v*.*2.7.1 program [5] and processed by Blast2go software (Table 2) [6], [7], [8]. An overview of *Myristica fatua* Gene Ontology (GO) classification is presented in Fig. 1a for Biological Process, Fig. 1b for Molecular Function, and Fig. 1c for Cellular Component [9] and KEGG pathways [10]. Open reading frames (ORFs) from transcripts were determined using the TransDecoder program (Table 3) [11]. The distribution of the identified EST-SSRs in transcripts was performed using the MISA program (Table 4) [12].

**Table 1:** Read and assembly statistics of *Myristica fatua* leaves.

Features	Numbers
Number of reads	4,379,800
Number of bases	3,467,934,331
Mean read length (bp) and quality	791.8 / 12.2
Read length N50 (bp)	1,010
Number and bases total (bp) of transcripts	21,098/ 34,073,111
Length range, average (bp), and N50 (bp) of transcripts	207 – 14,174 / 1,614.99 / 2,017

**Table 2:** Functional annotation summary of *Myristica fatua* transcripts.

Database Source	Number of transcripts (percentage)
- UniProt	17,038 (81%)
- GO Mapping	2,654 (13%)
- GO Annotation	11,004 (52%)
- GO EnzymeCode	5,351 (25%)
- KEGG	130 pathways

**Table 3:** Open Reading Frames (ORFs) prediction characteristics of *Myristica fatua* transcripts.

Features	transcripts Number (percentage)
- ORF transcripts	22,665
- ORFs Type:	
a. 5′prime_partial	3,855 (17.01%)
b. 3′prime_partial	684 (3.02%)
c. Internal	64 (0.28%)
d. Complete	18,062 (79.69%)

**Table 4:** Number of microsatellite regions observed in *Myristica fatua* transcripts.

Characteristics	Number
- Total number of identified SSRs	8,445
- Number of SSR containing transcripts	6,644
- transcripts containing more than 1 SSR	1,175
- SSRs present in compound motif	1,015
Motif	
- Dinucleotide	7,034
- Trinucleotide	1,285
- Tetranucleotide	1,194
- Pentanucleotide	81
- Hexanucleotide	16
- Heptanucleotide	29

## 2 Experimental Design, Materials and Methods

### 2.1 Sample collection

*Myristica fatua* leaves were collected from Karmanah's garden (S6°33′15.372′' E106°42′19.734′', Bogor, West Java, Indonesia).

### 2.2 Total RNA extraction

The total RNA from young leaves was extracted using the RNeasy PowerPlant Kit (Qiagen) following the manufacturer's protocol. The quality and quantity of RNA were checked by Nanophotometer NP-80 (Implen) and Qubit™ RNA Broad Range (BR) assay on Qubit® Fluorometer (Invitrogen).

### 2.3 Transcriptome sequencing and De novo assembly

The total RNA was subjected to RNA sequencing using PCR-cDNA Barcoding - SQK-PCB109 (PCB_9092_v109_revB_10Oct2019). The sequencing was performed on a Flow Cell R9.4.1 (FLO-MIN106D) on MinION Mk1B. After sequencing, the raw reads were base called using Guppy 6.1.2 with default parameters. Next, data pre-processing followed https://github.com/felixgrunberger/microbepore protocol includes demultiplexing and NanoStat v1.2.1 to assess the reads quality and reads' statistics [13]. Next, full-length reads with remaining SSP (strand-switching primer) and VNP (oligo-dT30VN) primers were identified using pychopper v2.5.0 (https://github.com/nanoporetech/pychopper). Then, polyA-tails and the remaining SSP adapters were removed using Cutadapt [14]. De novo assembly was then performed on clean full-length reads using the RATTLE program [15] (Table 1).

### 2.4 Functional annotation

The full-length polished transcripts were BLASTed using BlastX [16] with a cut-off of 10^−5^ using the filtered-UNIPROT database (Magnoliopsida (TaxID: 3398), downloaded on 19 October 2021). The blasted output was performed using Blast2Go software [17]. Open reading frames (ORFs) transcripts were predicted by the TransDecoder (https://github.com/TransDecoder/TransDecoder) with default parameters [18]. Microsatellite regions were observed using MISA software [19] (http://pgrc.ipk-gatersleben.de/misa) with modified parameters [20].

## Ethics statements

Not applicable.

## CRediT author statement

**Deden Derajat Matra**: Conceptualization, Methodology, Writing - Original Draft, Funding acquisition, **M Adrian**: Validation, Investigation, **Karmanah**: Resources, Writing - Review & Editing, **Jakty Kusuma**: Data Curation, Writing - Review & Editing, **Jérôme Duminil**: Conceptualization, Writing - Review & Editing, **Sobi**r: Supervision, Funding acquisition, **Roedhy Poerwanto**: Supervision, Conceptualization, Funding acquisition.

## Declaration of Competing Interest

The authors declare that they have no known competing financial interests or personal relationships that could have appeared to influence the work reported in this paper.

## Data Availability

RujakBase project - Myristica database for Whole Genome and Transcriptome Studies (Original data) (European Nucleotide Archive (ENA)). RujakBase project - Myristica database for Whole Genome and Transcriptome Studies (Original data) (European Nucleotide Archive (ENA)).
